# Anticancer Efficacy of 6-Gingerol with Paclitaxel against Wild Type of Human Breast Adenocarcinoma

**DOI:** 10.3390/molecules27092693

**Published:** 2022-04-22

**Authors:** Kamila Wala, Wojciech Szlasa, Natalia Sauer, Paulina Kasperkiewicz-Wasilewska, Anna Szewczyk, Jolanta Saczko, Nina Rembiałkowska, Julita Kulbacka, Dagmara Baczyńska

**Affiliations:** 1 Department of Dermatology, Venereology and Allergology, Wroclaw Medical University, 50-368 Wroclaw, Poland; kamila.wala.01@gmail.com; 2 Faculty of Medicine, Wroclaw Medical University, 50-368 Wroclaw, Poland; wojciech.szlasa@student.umw.edu.pl; 3 Faculty of Pharmacy, Wroclaw Medical University, 50-368 Wroclaw, Poland; natalia.sauer@student.umw.edu.pl; 4 Department of Chemical Biology and Bioimaging, Faculty of Chemistry, Wroclaw University of Science and Technology, 50-368 Wroclaw, Poland; paulina.kasperkiewicz-wasilewska@pwr.edu.pl; 5 Department of Molecular and Cellular Biology, Faculty of Pharmacy, Wroclaw Medical University, 50-368 Wroclaw, Poland; a.szewczyk@umw.edu.pl (A.S.); jolanta.saczko@umw.edu.pl (J.S.); nina.rembialkowska@umw.edu.pl (N.R.); dagmara.baczynska@umw.edu.pl (D.B.); 6 Department of Animal Developmental Biology, Institute of Experimental Biology, University of Wroclaw, 50-368 Wroclaw, Poland

**Keywords:** 6-gingerol, paclitaxel, breast cancer, anticancer therapy, cytostatic drug

## Abstract

Breast cancer is one of the most common malignant neoplasms, and despite the dynamic development of anticancer therapies, 5-year survival in the metastatic stage is still less than 30%. 6-Gingerol (1-[4′-hydroxy-3′-methoxyphenyl]-5-hydroxy-3-decanone) is a substance contained in ginger, which exhibits anti-cancer properties. Paclitaxel is a cytostatic substance used to treat breast cancer, but its therapeutically effective dose has many adverse effects. The aim of the presented study was to assess the anticancer effect of 6-gingerol and the possibility of increasing the effectiveness of Paclitaxel in the death induction of wild type human breast cancer cells. MCF-7/WT cells were treated with drugs—6-gingerol and paclitaxel at selected concentrations. The mitochondrial activity assay, caspase 7 activity assay, ATP assay, microscopy studies, and RT-PCR assays were performed to evaluate the antitumor activity and mechanism of action of both compounds, alone and in combination. After 72 h of incubation, the mitochondrial activity showed that the combination of 5 nM Paclitaxel with 10 µM 6-Gingerol led to the same decrease in viability as the use of 20 nM Paclitaxel alone; 10 µM 6-Gingerol led to an enhancement of caspase 7 activity, with the highest activity observed after 24 h of incubation. A real-time PCR study showed that 6-Gingerol induces the simultaneous transcription of Bax with TP53 genes in large excess to BCL-2. In contrast, 5 nM Paclitaxel induces TP53 transcription in excess of BCL-2 and Bax. Our results suggest that 6-Gingerol may act as a cell death-inducing agent in cancer cells and, in combination with paclitaxel, and increase the effectiveness of conventional chemotherapy.

## 1. Introduction

Breast cancer is one of the most common malignancies in the world [1]. In 2021 alone, more than 280,000 cases of breast cancer among women were diagnosed in the United States, and approximately 40,000 died from this cancer. Treatment outcomes and prognosis depend primarily on the stage of the disease at the time of diagnosis. Despite the dynamic development of anticancer therapies, 5-year survival in the metastatic stage is still less than 30% [2]. Importantly, the analysis of the 25-year period of the global trend shows that the incidence and prevalence of this cancer are significantly increasing [3]. The primary treatment option is surgical resection of the tumor, in the case of breast conserving surgery followed by radiation therapy. In more advanced stages, adjuvant chemotherapy or hormone therapy is necessary [4]. Currently, targeted therapy and immunotherapy have been gaining importance. However, systemic treatment is associated with numerous side effects [5]. Additionally, the effects are often insufficient to obtain full remission because of resistance to the therapy. According to the literature, primary resistance to systemic therapy is reported in 20–40% of breast cancer cases [6,7]. 

In recent years, much attention has been paid to agents of natural origin that alone or in combination with commonly used therapy, can reduce the risk of the adverse effects of chemotherapy and at the same time increase their anti-cancer effect. Some of them show a multidirectional action, including antioxidant, cytostatic or cytotoxic properties [8]. 6-Gingerol (1-[4′-hydroxy-3′-methoxyphenyl]-5-hydroxy-3-decanone) is a phenolic phytochemical found in ginger and is one of its most pharmacologically active compounds [9]. Numerous studies prove that 6-Gingerol exhibits antioxidant, anti-inflammatory, antiplatelet, antimicrobial (including viruses, such as Covid-19) as well as anticancer properties [10,11,12,13]. The antitumor effect is obtained by influencing various metabolic pathways of neoplastic cells, including cell cycle regulation, angiogenesis inhibition and activation of apoptosis [14]. It was observed that 6-gingerol influences the cell cycle by inhibiting the translation during protein biosynthesis pathway-cyclin-dependent kinases necessary for the proper course of G1 and G2 phases during cell division [14]. The pro-apoptotic effect has been demonstrated, among others, on ovarian cancer cell lines SKOV-3 and in a mouse skin cancer model [15,16]. In both cases, changes in p53 protein expression were noticed. Additionally, by inhibiting angiogenesis and reducing cell motility, 6-gingerol decreases the risk of metastasis [17,18]. Recently, the anti-tumor activity of 6-gingerol has also been demonstrated on breast cancer cell lines (MCF-7 and MDA-MB-231), with activation of cell cycle arrest, ROS production, and p53-dependent intrinsic apoptosis [19].

Paclitaxel is a cytostatic substance belonging to the group of taxanes. In medicine, it is used to treat cancers of the breast, ovary and non-small cell lung cancer [20]. Its antimitotic activity is based on the inhibition of depolymerization of microtubules necessary for the M phase of cell division [21]. However, its use in a therapeutically effective dose causes many adverse effects, including bone marrow suppression, neuropathies and gastrointestinal complaints [22]. 

The aim of the presented study was to evaluate the antitumor activity of 6-Gingerol in combination with Paclitaxel against breast cancer cells. Attempts have been made to determine how 6-gingerol interacts with a standard cytostatic drug and whether such a combination will allow the paclitaxel dose to be reduced while retaining the anti-cancer effect. For this purpose, a number of studies were carried out to assess the effects of the above-mentioned substances, alone and in combination, on, among others, the mitochondrial activity of cancer cells or the expression of certain proteins that regulates apoptosis. The obtained results will allow for a preliminary assessment of the effectiveness of 6-Gingerol as a potential agent that may enhance the effect of standard anti-cancer drugs in the treatment of breast cancer.

## 2. Results

### 2.1. MTT Viability Assay and ATP Cellular Content

**Figure 1:**
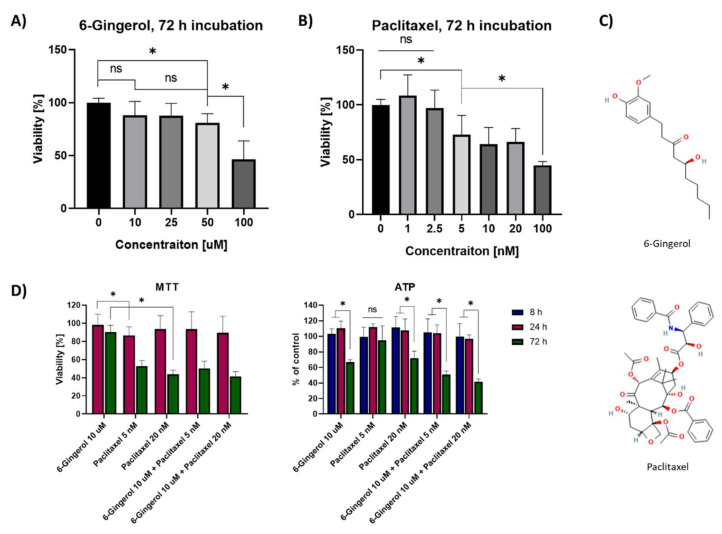
MTT viability assay 72 h after incubation with (**A**) 6-Gingerol and (**B**) Paclitaxel in concentrations ranging from 0 to 100 µM and from 0 to 100 nM, respectively. (**C**) Structures of 6-Gingerol and Paclitaxel; (**D**) MTT and ATP assays after a combination of 6-Gingerol with 5 nM and 20 nM Paclitaxel assessed after 8, 24 and 72 h, * *p* < 0.05 in the Kruskal–Wallis test for (**A**,**B**) or ANOVA test for (**D**); ns—not statistically significant.

Figure 1 presents the changes in the viability of MCF7 cells after 72 h incubation with 6-Gingerol (A) and Paclitaxel (B) in varying concentrations of both substances. Gingerol remains a natural compound with the ability to interact with cell membranes and paclitaxel is a cytostatic drug, which acts via the inhibition of the cell cycle and thus affects cell divisions (structures on in Figure 1C). The lowest concentration of 6-gingerol, which decreased the mitochondrial activity of breast cancer cells was 50 µM. The most pronounced drop in viability was observed between 50 and 100 µM, where the viability was estimated at 80.8 ± 6.6% and 46.5 ± 11.8%, respectively. In the case of Paclitaxel, there was increase in the mitochondrial activity of the cells to 108.4 ± 12.3% in the low 1 nM concentration of the drug. Conversely, between 2.5 and 5 nM concentrations of the cytostatic drug a decrease in the cells’ viability to 97.0 ± 11.5% and 72.7 ± 11.9%, respectively, was observed. As the concentration of Paclitaxel increased, a decrease in viability was noted (64.1 ± 10.3% for 10 nM; 66.4 ± 9.7% for 20 nM, 44.9 ± 3.3% for 100 nM), Interestingly, there was no statistical difference from 5 up to 20 nM of Paclitaxel. 

Our study aimed to investigate the effects of 6-Gingerol in non-cytotoxic concentration on the efficacy of Paclitaxel against breast cancer cells, thus for further studies, we chose 10 µM of 6-Gingerol and combined it with 5 and 20 nM Paclitaxel to find, whether we could decrease the effective dose of the latter drug. Figure 1D shows the effects of 10 µM 6-gingerol combination with Paclitaxel in 5 and 20 nM concentrations against MCF-7/WT cells. The MTT assay after 72 h showed no cytotoxic effect of 6-gingerol on the cells, but a high level of cell death after its combination with Paclitaxel (viability of 90.56 ± 7.3% with 6-Gingerol alone versus 50.3 ± 7.9% in combination with 5 nM Paclitaxel and 41.5 ± 5.4% with 20 nM Paclitaxel). The effect was not different from standalone Paclitaxel treatment, where the viability was 52.7 ± 6.4% for 5 nM and 43.7 ± 4.5% for 20 nM. Interestingly, after 72 h of incubation standalone 6-Gingerol decreased the ATP content by more than 5 nM Paclitaxel (66.7 ± 3.1% versus 95.0 ± 13.5%) with a similar effect to 20 nM Paclitaxel, where ATP content was 72.0 ± 8.8% of control (Figure 1D). Combined therapy with 6-gingerol and paclitaxel at a concentration of 5 nM or 20 nM led to a significant decrease in ATP to 50.6 ± 4.9% and 41.65 ± 3.5%, respectively. Even though the cells’ viability remains not affected by the combination of 6-gingerol with Paclitaxel, the differences in ATP content in the cell following incubation with the drugs prove that 6-gingerol might act on the cells by different mechanisms to standalone Paclitaxel.

### 2.2. Caspase-7 Activity Assay, Real Time PCR Study and Confocal Microscopy Studies

**Figure 2:**
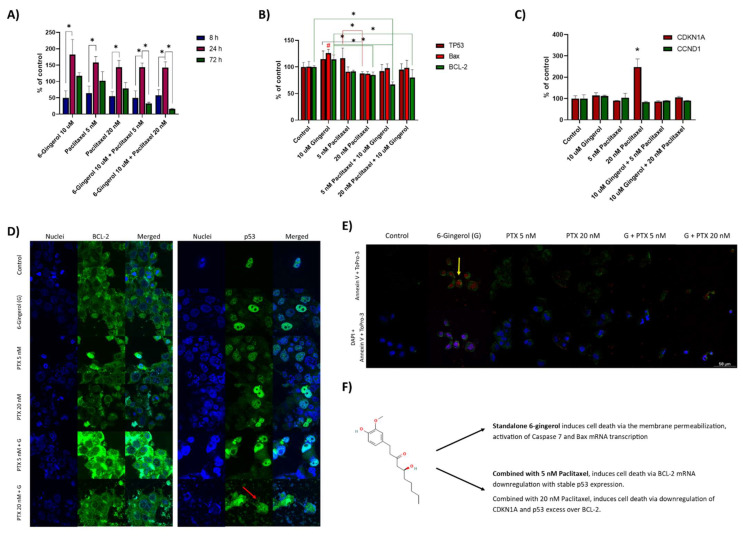
(**A**) Caspase-7 activity in MCF7-WT cells after the combination of 6-Gingerol with Paclitaxel. Data were captured after 8, 24 and 72 h following the start of incubation with the compounds. * *p* < 0.05 ANOVA; (**B**) RT-PCR analysis of TP53, Bax and BCL-2 mRNA in MCF-7/WT cells after 24 h incubation with 6-Gingerol and Paclitaxel. # *p* < 0.05; * *p* < 0.05 one-side ANOVA; (**C**) RT-PCR analysis of CDKN1A and CCND1 mRNA in MCF-7/WT cells after 24 h incubation with 6-Gingerol and Paclitaxel. * *p* < 0.05 ANOVA; (**D**) MCF-7/WT cells after incubation with the combination of 6-Gingerol with Paclitaxel-Cells morphology and localization of BCL-2 and p53 in confocal laser microscopy studies; (**E**) Annexin V and To-Pro-3 staining studies after 24 h incubation with the combination of 6-Gingerol with Paclitaxel. (**F**) Summary of the action of 6-gingerol standalone and in combination with paclitaxel on the MCF-7 cell line.

To analyze the mechanism of 6-gingerol combination with Paclitaxel, we analyzed the activation of caspase 7, mRNA level of TP53, Bax, and BCL-2. Moreover, we analyzed the effects of the combination therapy on cell cycle regulation via CDKN1A and CCND1. Figure 2A presents the Caspase 7 activity in the cells following the combinatorial incubation with 6-Gingerol and Paclitaxel. After 8 h incubation, no statistical changes were observed. After 24 h the activity of the caspase was elevated in all the possible samples, to 182 ± 36.5% after incubation with 10 µM 6-gingerol and to about 150% in the rest of the samples. In contrast, after 72 h of incubation, we captured that the combination of 5 nM Paclitaxel with 10 µM 6-Gingerol leads to a stable decrease of caspase activity with the increasing concentrations of the drugs—32 ± 4.0% for 5 nM and 16 ± 1.6% for 20 nM Paclitaxel. Caspase 7 activity is most pronounced after incubation with 10 µM 6-Gingerol. The activity decreases to the control level (117 ± 9.9%) after 72 h incubation with 6-Gingerol and corresponds to the cells’ viability after treatment with other compounds. 

Figure 2B presents the Real Time PCR study of the mRNA content in the cells following 24 h incubation with 6-Gingerol and Paclitaxel. The study reveals that 5 nM Paclitaxel induces the transcription of TP53 in excess to the BCL-2 and Bax (116.5 ± 14.1% versus 91.6 ± 2.4% and 91.2 ± 9.0% of control). Therefore, the cells prepare for cell repair and do not induce apoptosis. Curiously, the addition of 6-Gingerol induces the simultaneous transcription of Bax (126.2 ± 6.9% of control; # *p* < 0.05) in high excess over the BCL-2 (114.4 ± 8.3%. Therefore, the mechanism in which 6-Gingerol acts on the MCF-7/WT cells includes the induction of proapoptotic Bax transcription, which is not present after standalone incubation with 5 nM Paclitaxel. Interestingly, confocal microscopy staining studies revealed that the combination of 5 nM Paclitaxel with 10 µM 6-gingerol leads to the overexpression of antiapoptotic BCL-2 protein. In this way the cell prevents cell death for a short time, but the lowered level of BCL-2 mRNA (66.9 ± 5.3% of control) leads to an advantage of the proapoptotic effect in the 72 h viability assay (refer to Figure 1D). In contrast, cells treated with 20 nM Paclitaxel combined with 10 µM 6-gingerol did not overexpress BCL-2, however, its mRNA decreased to 80.0 ± 14.6%. Interestingly, cells treated with 6-gingerol underwent cell damage in morphological study of the cells’ integrity. Figure 2C shows the level of mRNA for cyclin dependent kinase N1A and cyclin D1 after treatment of MCF-7/WT cells with the drugs. Only 20 nM paclitaxel induces the excessive transcription of CDKN1A mRNA, which increased by approximately 150% after 24 h incubation with the drug (248.2 ± 37.8%, * *p* < 0.05). Noticeably, the combination of 6-gingerol with 20 nM Paclitaxel does not lead to the same level of CDKN1A transcription (106 ± 4.6% of control), thus the combination of both drugs acts differently to the standalone 20 nM paclitaxel, even though the viability of the cells following treatment with the drugs remains similar. 

To validate our findings, we performed staining studies of dead cells with To-Pro-3 dead cell stain and apoptotic cells with annexin V (Figure 2E). We can see that after incubation with 6-gingerol, the cells remain permeable towards To-Pro-3 even though they have almost 100% viability (refer to Figure 1D). Except for control sample, all the cells treated with both standalone drugs and with their combination presented a fluorescence of annexin V, and therefore, undergo apoptosis, which is compatible with caspase 7 activation studies after the same (24 h) incubation time (Figure 2A). Figure 2E summarizes the effects of 6-gingerol on MCF-7/WT cells and in combination with Paclitaxel. We can observe, that the standalone 6-gingerol induces membrane permeabilization to To-Pro-3 with nearly no effect on cells’ viability after 24 h. Moreover, the drug induces the activation of caspase 7 and Bax transcription. The cytotoxic effect is observed, however, when gingerol is combined with paclitaxel. In this case, the cycle-inhibiting drug does not work in this way anymore and the BCL-2/p53 equilibrium leads to cell cytotoxicity. 

## 3. Discussion

The anti-cancer potency of 6-Gingerol has been proven for many types of cancer cells including colorectal cancer, ovarian cancer, cervical cancer and retinoblastoma. In the experiments described in the literature, the most frequently used concentrations were in the range of 1–100 µM [23,24,25,26,27]. In our study, the minimum concentration of 6-Gingerol at which a statistically significant decrease in mitochondrial activity was observed was 25 µM. As the concentration increased, a further decrease in mitochondrial activity was observed with cell viability < 50% at a concentration of 100 μM. Additionally, other studies confirmed the dependence of the cytotoxic response on the concentration of the 6-gingerol extract used [15]. The molecular mechanism of action of 6-gingerol described in the literature is most often based on the inhibition of cell cycles and the induction of apoptosis. The phase at which the cycle is stopped is different for each cell line. In the case of HeLa cells, the use of 6-gingerol is followed by G2-phase arrest, in SCC4 cells S-phase cessation [26]. Dose dependent G2/M phase cell cycle arrest was seen in retinoblastoma cells (RB355) and in colon cancer cells (LoVo) as a result of the reduction of cyclin (A, B1) and cyclin-dependent kinases upon exposure of cells to 6-gingerol [24]. Interestingly, we noticed that the level of mRNA for cyclin dependent kinase N1A and cyclin D1 after treatment of MCF-7/WT cells with 10 µM 6-gingerol remained unchanged. Only 20 nM paclitaxel inhibits the cell cycle by increasing the level of CDKN1A mRNA. In the previously mentioned studies, significant G2/M phase arrest of Rb355 cells was seen at the concentration of 75 and 150 µM. This suggests that low concentrations of 6-gingerol (10 µM) potentiate the anti-tumor effects of paclitaxel, but this effect is not based on inhibition of the cell cycle. Another mechanism of action involving the induction of apoptosis may be the consequence of caspase activation. In our study on MCF-7/WT cells, it was found that 6-gingerol increased caspase-7 activity with the greatest enhancement being seen after 24 h, with a decrease to the control level after 72 h incubation. Caspase-3 activation was observed also in human cervical or oral cell lines (HeLa, SCC4) after 36 h of incubation with 6-gingerol [26]. In a study on the MCF-7 cell line, gene expression analysis showed a significant increase in the expression of caspases, including caspase-3 and caspase-8. The most significant growth was seen for caspase-3, where expression increased over 6-fold [28].

Bcl-2 family proteins are responsible for the regulation of programmed cell death. Some molecules, such as Bcl-2 and Bcl-XL, inhibit apoptosis, while Bax promotes cell death. The expression of Bax is downregulated in tumor cells, such as colorectal cancer cells or ovarian cancer cells, which is associated with poorer treatment response to 5-fluorouracil and cisplatin [29]. The activation of apoptosis by 6-gingerol was shown to be dependent on the mitochondrial pathway. This anticancer agent promotes apoptosis by increasing both the expression of the Bax gene and the Bax/Bcl-2 ratio. In our study on MCF-7/WT cells, RT-PCR analysis revealed that 6-gingerol primarily influenced BAX transcription and this enhancement was in high excess over BCL-2. Such an effect does not occur after incubation with 5 nM Paclitaxel, where the induction of p53 mRNA synthesis prevails, so the cell tries to repair the damage and does not induce apoptosis. Similarly, in the experiment of Zhang et al. in human cervical adenocarcinoma cells the Bax/Bcl-2 ratio was increased after incubation with 6-gingerol [30]. Comparable results were obtained in the study by Nipin et al., which was carried out on the MCF-7 and MDA-MB-231 cell lines. The increase in Bax protein expression was more intense than in the presented research, however, in the cited studies, several times higher concentrations of 6-gingerol (100–200 µM) were used [19]. 

In the literature, the described in vitro and in vivo studies show that 6-gingerol also modifies pathways of cell signaling, acting on other molecules, such as Mitogen-Activated Protein Kinases (MAPK), Nuclear Factors (NF-κB), ERK1/2/JNK/AP-1 pathway, or pro-inflammatory mediators (TNF-α and COX-2) [12,31,32]. Moreover, 6-Gingerol has anti-cancer properties at many stages of tumor development, including the spread of cancer cells. Lee et al. proved the effectiveness of 6-gingerol at the concentration of 10 µM in reducing the synthesis and activity of metalloproteinases (MMP-2 and MMP-9), which inhibits the adhesion of breast cancer cells (MDA-MB-231) and may reduce metastasis. At the lower concentration (5 µM), no similar changes were observed [18]. Similar results were obtained by Zhao et al. After 24 h incubation with 6-gingerol (also at 10 μM) the expression of N-cadherin, MMP-2 and MMP-9 declined in human HPV-positive cervical cancer cells (HeLa) [33]. Although in our study we did not investigate the influence of 6-gingerol on the synthesis of metalloproteinases or proteins of other metabolic pathways, we noticed a different effect of 6-gingerol on cancer cells than previously described in the literature. Namely, cells treated with 6-gingerol underwent cell damage in a morphological study of the cells’ integrity. Staining studies of dead cells with To-Pro-3 dead cell stain and apoptotic cells with annexin V confirmed that the standalone 6-gingerol induces membrane permeabilization to To-Pro-3 with nearly no effect on cell viability after 24 h, which was also previously shown in MTT studies (almost 100% viability after 24 h incubation with 10 µM 6-gingerol). However, the cytotoxic effect is observed, when gingerol is combined with paclitaxel.

Another essential property of gingerol is the enhancement of the efficacy of conventional anticancer drugs. When assessing the mitochondrial activity, we proved that with the use of 10 µM of 6-gingerol it is possible to reduce the dose of paclitaxel up to four times to achieve a similar therapeutic effect. Zhang et al. showed that 6-gingerol works synergistically also with other classic anticancer drugs, increasing the cytotoxicity of, e.g., 5-fluorouracil (5-FU) and paclitaxel against the HeLa cervical cancer cell line. The synergism of 6-gingerol with 5-FU is so significant that it allows reducing the dose of 5-FU used by half. On the other hand, in the case of combining 6-gingerol with Ptx, even a 9-fold increase in the dose of Ptx in monotherapy does not allow to achieve similar results as in the case of combined therapy [30]. In both the standalone and combination therapy with 6-Gingerol and Paclitaxel, we noticed a significant difference in cell viability between 24 and 72 h of incubation. An aspect worth further consideration is the trend of changes in cell viability depending on the time of incubation with drugs. Literature data allow us to conclude that the cytotoxicity of both Paclitaxel and 6-gingerol towards MCF-7 cells changes with the increase of exposure time to these drugs in a gradual manner [34,35]. Considering the significant reduction of mitochondrial activity after the combination therapy of 6-gingerol with paclitaxel, the manner of interaction of these agents should be further investigated. Our study did not evaluate the combination index (CI), although the results may suggest synergistic effects of both substances. Zhang et al. has already proven that 6-gingerol at all tested concentrations (from 5 to 45 µM) shows a significant synergistic effect on Hela cells in combination with Paclitaxel or 5-fluorouracil. Additionally, in the case of therapy with Paclitaxel, the CI value did not exceed 0.5, which indicates a strong synergism [30]. 6-gingerol also shows a synergistic effect in combination with Doxorubicin within HepG2 and Huh-7 cells (CI < 0.3) and with γ-Tocotrienol in HT-29 and SW837 Human Colorectal Cancer Cells (CI < 0.9) [36,37]. However, in the study on glioblastoma cells, after combining 6-Gingerol with Tocotrienol in only one cell line (LN18), a synergistic effect was obtained (CI = 0.8). In the remaining two cell lines, 1321N1 and SW1783, the CI was 1.24 and 1.29, respectively, which indicated an antagonistic effect of the substances used [38]. The studies cited above show that in order to accurately determine whether the effect of combining 6-gingerol with Paclitaxel on MCF-7 cells is synergistic or additive, further studies on this cell line must be carried out.

In another study, 6-gingerol at a concentration of 50 µM increases the cytotoxicity of cisplatin in the treatment of cervical cancer (HeLa cells) by increasing the accumulation of cells in the G2/M phase [39]. Our results show that only 20 nM paclitaxel induces excessive transcription of CDKN1A mRNA after 24 h of drug incubation. However, 6-gingerol alone or 6-gingerol with 20 nM paclitaxel does not lead to the same level of CDKN1A transcription, so the combination of the two drugs works differently from 20 nM paclitaxel alone. As shown before, 6-Gingerol promotes apoptosis by activation of caspase 7 and Bax transcription. This effect was confirmed in RT-PCR studies, in which the addition of 6-gingerol to paclitaxel increased the BAX/Bcl-2 ratio. These results suggest that the cytostatic drug in combination therapy does not act on the principle of cell cycle arrest but leads to cellular cytotoxicity via activating the mitochondrial pathways leading to apoptosis. Moreover, Dae-Hee Lee et al. established that 6-gingerol, by enhancing TRAIL-induced apoptosis in tumor cells with overexpression of anti-apoptotic proteins, may also contribute to the reduction of drug resistance of tumor cells, and thus indirectly enhance the effect of anti-tumor agents [40].

The results of our research confirm the possibility of using 6-gingerol to enhance the anti-cancer effect of conventional cytostatic drugs. When considering the potential use of this substance in further research, including clinical trials, pharmacokinetics should also be taken into account. 6-gingerol is characterized by low water solubility, therefore, for in vitro tests this agent should be dissolved in DMSO or ethanol. To achieve the appropriate tissue concentrations in in vivo tests, Wang et al. designed and tested pro-liposomes containing 6-gingerol, which increased 5-fold the bioavailability of this compound after oral administration in in vivo studies. Interestingly, the liposomal formulation of 6-gingerol at the same time showed a greater anti-tumor effect [41]. Given the potential to increase bioavailability and reduce side effects of cancer therapy through the use of nanotechnology solutions, this field seems to be a promising target for further research on the use of 6-gingerol in oncology.

## 4. Materials and Methods

### 4.1. Cell Culture

An MCF-7/WT cell line, derived from a 69-year-old Caucasian female with metastatic breast cancer, was selected for this study. Cells were a kind gift from the Department of Tumor Biology, Comprehensive Cancer Center, Maria Sklodowska-Curie Memorial Institute in Gliwice (Poland). The cells were cultured in growth medium: Dulbecco’s modified Eagle’s medium (DMEM, Sigma-Aldrich, St. Louis, MO, USA) supplemented with 10% fetal bovine serum (FBS, Sigma-Aldrich, St. Louis, MO, USA), 1% glutamine, and antibiotics (penicillin/streptomycin, Sigma-Aldrich, St. Louis, Missouri, USA) under standard culture conditions at 37 °C, in a 5% CO_2_ humidified atmosphere. For the experiments, the cells were rinsed with PBS and removed by trypsinization (trypsin 0.025% and EDTA 0.02%).

### 4.2. Preparation of Drug Solutions

6-Gingerol (ab145635) and Paclitaxel (ab120143) were obtained from Abcam (Cambridge, UK). Both reagents were dissolved according to the manufacturer’s protocols. DMSO was used as a solvent. Then, immediately before the tests, the substances were diluted in DMEM to obtain the concentrations necessary for MTT assays. 6-Gingerol was diluted to concentrations of 10, 25, 50 and 100 µM and paclitaxel to a concentration of 1, 2.5, 5, 20, 50 and 100 nM. For further studies, 6-gingerol solution at a concentration of 10 µM and paclitaxel at a concentration of 5 and 20 nM were used. DMEM without drugs was used as a control.

### 4.3. MTT Viability Assay

Cells’ viability was analyzed with mitochondrial activity assay (MTT). The culture medium was removed from each of the wells, and 100 μL of 0.5 mg/mL MTT (3-(4,5-dimethylthiazol-2-yl)-2,5-diphenyltetrazolium bromide, Sigma-Aldrich) solution in PBS buffer was added. After 1.5 h of incubation at 37 °C, acidified isopropanol (100 μL, 0.04 M HCl in 99.9% isopropanol) was added to dissolve the formazan crystals. The samples were fully dissolved by the pipet mixing technique. The absorbance was measured at 560 nm using a multiplate reader (GloMax, Promega, Madison, WI, USA). The results were expressed as the percentage of viable cells relative to untreated control cells.

### 4.4. Caspase 7 Activity Assay

The activity of caspase 7 in MCF-7/WT cells was measured with the use of the Caspase-Glo 3/7 Assay System (Promega, Madison, WI, USA). Cells were seeded in 96-well plates. After incubation with the appropriate anticancer agents (6-gingerol at a concentration of 10 µM and paclitaxel at a concentration of 5 and 20 nM, separately and in combination), 100 µL of Caspase-Glo 3/7 reagent was added to each well and the cells were incubated at room temperature for 1 h. The luminescent signal was measured with a GloMax Reader (Promega, Madison, WI, USA). Results are expressed as the percentage of cell caspase 7 activity relative to the untreated control cells.

### 4.5. CellTiter Glo ATP Assay

Cells were seeded in 96-well plates and allowed to grow for 24 h. Subsequently, cells were incubated with anticancer agents in appropriate concentrations (6-gingerol at a concentration of 10 µM and paclitaxel at a concentration of 5 and 20 nM), for 8, 24, or 72 h. The ATP content was assessed using the CellTiter-Glo (Promega, Madison, WI, USA) luminescence test according to the manufacturer’s instructions. Luminescence was measured with a microplate reader directly from the 96-well plates.

### 4.6. Confocal Microscopy Studies

BCL-2 (ab59348, Abcam, Cambridge, MA, USA) and p53 (ab154036, Abcam, Cambridge, MA, USA) staining were performed to evaluate protein expression in the MCF-7/WT cell line following the incubation with 6-gingerol and paclitaxel. The cells were seeded on the cover glasses in 6-well plates overnight to attach. Then, 24 h or 72 h incubation with anticancer drugs: 6-gingerol at a concentration of 10 µM and paclitaxel at a concentration of 5 and 20 nM, was performed. After that, cells were fixed in a 4% formalin solution. The samples were permeabilized with Triton-X100 for 5 min and incubated with FBS concentration for 1 h. Following this, the cells were washed with Triton-X100, and primary antibody dilution was added for 1 h incubation. The cells were rinsed with PBS, and the secondary antibody was added for 1 h incubation. Once again, the samples were rinsed with PBS. FluorshieldTM with DAPI (4,6-diamidino-2-phenylindole) was applied to visualize the nuclei and mount the cells. The samples after 72 h of incubation were observed on the Olympus FluoView FV1000 confocal laser scanning microscope (Olympus, Tokyo, Japan).

Similarly, we performed the Annexin V and To-Pro-3 staining studies. After 24 h of incubation with the drugs, we stained the unfixed cells with the mentioned stains. We incubated the cells attached to the cover glasses for 30 min and afterward, we rinsed the glasses with PBS three times at RT. In the end, we fixed the cells and visualized them under the confocal laser microscope (Leica TCS SP8 LSCM, Mannheim, Germany), with 488 nm laser (for Annexin-V), and 648 nm laser (for To-Pro-3). 

### 4.7. Real-Time PCR 

Cells were seeded on Petri dishes and then incubated with anticancer agents (6-gingerol 10 µM, paclitaxel 5 nM and 20 nM, separately and in combination) in the culture medium for 24 h. After that, the cells were collected and dry cell pellets were stored at −20 °C for further experiments. The total RNA was isolated using a NucleoSpin RNA II kit (Macherey-Nagel & Co., Düren, Germany) following the manufacturer’s protocol. Reverse transcription reaction (RT) was performed using 500 ng of extracted total RNA and a High-Capacity cDNA Reverse Transcription Kit (Thermo Fisher Scientific, Waltham, MA, USA) in a final volume of 20 μL according to the manufacturer’s instruction. AceQ qPCR Probe Master Mix (Vazyme Biotech, Nanjing, China) and specific TaqMan Assays: Hs00180269_m1 (for BAX), Hs00608123_m1 (BCL-2), Hs01034249_m1(TP53), and Hs99999905_m1 (for glyceraldehyde-3-phosphate dehydrogenase; GAPDH) (Thermo Fisher Scientific Waltham, MA, USA) were used to assess RNA expression according to the manufacturers’ instructions. We added 3 μL of three-times-diluted RT products to a single real-time polymerase chain reaction (RT-PCR). All the reactions were performed in triplicate in 96-well plates under the following thermal cycling conditions: 5 min at 95 °C followed by 40 cycles of 10 s at 95 °C and 30 s at 60 °C. The reactions were run in the Optical Real-Time PCR Thermocycler (Biometra GmbH, Göttingen, Germany) and the threshold cycle data (Ct) were collected using qPCRsoft (Biometra GmbH, Göttingen, Germany). For relative quantification (RQ), the samples were normalized against the expression of GAPDH mRNA using 2^−ΔΔCT^ method.

### 4.8. Statistical Analysis

The experiments were performed in 3 replicates. A normality test using The Kolmogorov–Smirnov test method was performed and when the data passed the normality test, we used ANOVA statistics. Alternatively, the Kruskal–Wallis test was used for non-normal distribution. Data were expressed as mean ± SD and analyzed by one-way ANOVA followed by Tukey post-hoc test (in GraphPad Prism 8), with *p* < 0.05 being considered statistically significant.

## 5. Conclusions

The performed study indicates that 6-gingerol is a promising supportive anticancer agent when combined with a cytostatic drug. Moreover, 6-gingerol induced the activation of caspase 7 and Bax transcription. However, the most significant cytotoxic effect was noted when gingerol was combined with paclitaxel. Standalone 6-gingerol induced membrane permeabilization to To-Pro-3 dye with nearly no effect on cell viability after 24 h. This sensitizing effect of the lipid membrane can be used to improve drug delivery, and then the cell membrane is more permeable to drug molecules. In this case, the cycle-inhibiting drug does not work in this way anymore and the BCL-2/p53 equilibrium leads to cell cytotoxicity. This study suggests that 6-gingerol might be effectively used for the development of antitumor protocols against breast adenocarcinoma. 

## Data Availability

Not applicable.

## Sample Availability

Not available.
